# Preface from the Guest Editor of Special Issue “Simple Substances of Non-Metals: Molecular Structures Modeling with Using DFT and More Advanced Methods of Quantum Chemistry”

**DOI:** 10.3390/ijms22020815

**Published:** 2021-01-15

**Authors:** Oleg V. Mikhailov

**Affiliations:** Department of Analytical Chemistry, Certification and Quality Management, Kazan National Research Technological University, K. Marx Street 68, 420015 Kazan, Russia; olegmkhlv@gmail.com

According to the traditional definition of the concept of “simple substance” adopted in chemistry, it is as a chemical compound, the structural unit of which (molecule, ion, radical, the link of the polymer chain, the unit cell of the crystal lattice, etc.) includes atoms (more precisely, nuclides) and only one chemical element. The number of atoms in the structural unit of the substance is not limited by anything and it can therefore include several units (two in the molecules of molecular nitrogen N_2_ and oxygen O_2_, four in the molecules of white phosphorus P_4_ and yellow arsenic As_4_, eight in molecules of the so-called orthorhombic sulfur S_8_), several tens of units (in molecules of C_60_, C_70_, and C_80_ fullerenes), several hundreds of units (in molecules of fullerene C_540_), to an infinitely large number (in high-molecular-weight simple substances like black phosphorus, graphite, diamond, carbyne, and, also, any metal). Regarding this connection, it should be noted that many of these, by definition, “simple substances” actually do not have a simple structure and, in terms of their quantitative composition, often turn out to be much more complicated than many so-called “complex substances” (which, according to the generally accepted definition, include substances whose structural units contain nuclides of two or more different chemical elements). For example, the most stable allotropic modification of the first of the p-elements, boron, namely β-boron, contains a carcass of twelve B_12_ atoms as a key structural fragment, in which boron atoms are located at the vertices of the most highly symmetrical of all polyhedra, namely, the icosahedron. Because simple substances, by definition, are formed by atoms of only one chemical element, between them, as a rule, two types of chemical bonds are realized—either atomic (covalent) or metallic, the first of which is characteristic of atoms of p-elements, which have sufficiently high electronegativity, and the second of atom elements with low and middle electronegativity. Taking this circumstance into account, two types of chemical elements forming simple substances—non-metals and metals—are often distinguished.

Despite its simple stoichiometric composition, the phenomenon of allotropy is very characteristic of simple substances: the same chemical element can form simple substances with different physical and chemical properties (so-called allotropic modifications). As a rule, the phenomenon of allotropy is caused either by the different composition of molecules of a simple substance (allotropy of composition) or by the specifics of the arrangement of its atoms and/or molecules in the crystal lattice (allotropy of the form). Therefore, these modifications may differ from each other in terms of their stoichiometric composition (these are, in particular, allotropic modifications of molecular oxygen—dioxygen O_2_ and trioxygen (ozone) O_3_, and the above-mentioned carbon modifications—fullerenes C_60_, C_70_ and C_80_), space structure with the same stoichiometric composition (iron modifications—ferrite with a body-centered cubic lattice and austenite with a face-centered cubic lattice, and high-molecular phosphorus modifications—red phosphorus and black phosphorus), and the external form of crystals (rhombic and monoclinic sulfur S_8_). However, a unique case is seen when the phenomenon of allotropy is associated neither with a difference in chemical composition, nor with a difference in the structure of structural units or external form; this situation occurs in the case of allotropic modifications to the simplest of all simple substances—molecular hydrogen H_2_—namely in orthohydrogen, in which the nuclear spins are parallel to each other, and parahydrogen, where these spins are antiparallel. For many years there have been heated debates about the possibility of hydrogen formation in a simple substance with metallic properties, and this question remains open to this day.

Be that as it may, currently, more than 400 varieties of simple substances are known. The ability of a chemical element to form allotropic forms is due to the structure of its atom, which determines the type of chemical bond, and the structure of molecules and crystals. A very important factor here is the so-called catenation—the ability of atoms of a chemical element to form homochain structures (for example, in the case of allotropic modifications of sulfur). The undisputed leader among chemical elements in terms of the number of formed allotropic modifications is carbon, which is the only chemical element in which the number of valence orbitals, the number of valence electrons and the maximum possible coordination number coincide with each other, and which, precisely due to this unique circumstance, can form long homochain structures containing many hundreds and even thousands of atoms. It is no coincidence that, for this element, in addition to the already mentioned graphite, diamond, carbyne and numerous fullerenes, about a dozen other types of simple substances are known, in particular, very diverse carbon nanotubes, nanofibers, glassy carbon, astralenes and amorphous carbon. In the first decade of the XXI century, a unique modification—graphene—was added to this list, the structure of which can be considered as one plane of layered graphite, separated from the bulk crystal. The discovery of this modification to carbon was so important that its authors, namely A.K. Geim and K.S. Novoselov, were awarded the highest award for a modern scientist—the Nobel Prize in Physics—in 2010 with the formulation «*for groundbreaking experiments regarding the two-dimensional material graphene*». Graphene, as can be seen from this formulation, was the first elementary two-dimensional crystal obtained; subsequently, simple substances with a similar structure were obtained based on other *p*-elements of IV (XIV) groups of the Periodic Table of Chemical Elements—silicene and germanene. The emergence of a fairly numerous assortment of fullerenes and nanotubes made it possible to speak of the formation of a new specific branch of chemical science at the junction of organic and inorganic chemistry—the physics-chemistry of fullerenes and nanotubes. The point is that, on the one hand, simple substances, according to a long-established tradition in chemistry, are objects of inorganic chemistry. On the other hand, according to the definition of the International Union of Pure and Applied Chemistry (IUPAC), any chemical compound containing at least one carbon–carbon bond in its structural unit is an organic compound, and, therefore, ALL currently known allotropic modifications of carbon should be considered (at least formally) as organic substances.

The tendency towards allotropy is more pronounced in non-metals than in metals. This is partly why simple substances formed precisely by *p*-elements-non-metals attracted more attention from scientists from different eras, long before the formation of chemistry as a science. Despite the relative simplicity of their chemical composition, they have a number of specific (sometimes unique) properties, due to which they have found many applications in various branches of science and practice of anthropogenic activity. Allotropic modifications to carbon include graphite and diamond (known already in ancient times, later making up the extreme points of the classical scale of hardness of substances), carbyne, fullerenes and nanotubes (which became known at the turn of the XX/XXI centuries and found application in semiconductor technology as photoresists, activators in the growth of diamond films, and in the creation of superconducting materials). The last known allotropic modification of carbon, graphene, is also not of purely academic interest: according to reliable estimates, it has high mechanical rigidity and high thermal conductivity. The high mobility of charge carriers in it, the highest among all known materials (at the same thickness), makes it a very promising material for use in a variety of applications, in particular as a future basis for nanoelectronics and a possible replacement for silicon in integrated circuits. The list of possible applications of allotropic modifications of simple substances can be continued.

Despite the fact that, to date, very impressive advances have been reached in terms of the physics and chemistry of simple substances, there are still many unresolved questions in this field of chemical science, such as the possibility of the existence of new, as yet unknown to science, polynuclear simple substances formed by both carbon and atoms of other *p*-elements, such as, nitrogen, phosphorus, oxygen and sulfur. In this regard, it is important to predict both the possibility of their existence and the determination of the quantitative parameters of the molecular and/or crystal structures that determine their physicochemical properties. This problem is currently being successfully solved due to the availability of both modern quantum–chemical calculation methods and computer technologies and the corresponding experimental technique. At the same time, there are still not enough theoretical works devoted to quantum–chemical calculations of the above simple substances by the DFT method and by more advanced methods, such as CCSD (T) and QCISD (T). In particular, a very interesting and important problem, both theoretically and practically, is the allotropy of polynuclear simple substances formed by nitrogen atoms (poly-nitrogens), for which there are a number of predictive studies, according to which the possibility of the existence of allotropic modifications of the given *p*-element corresponds to the number of atoms in the molecule, which ranges from 4 to 60. Evaluation of their thermodynamic characteristics shows that many of them, if obtained experimentally, could serve as components of high-energy fuel compositions. This Special Issue of the *International Journal of Molecular Sciences*, which includes articles and messages devoted to quantum–chemical calculations of the molecular structures of polynuclear simple substances formed by non-metal *p*-elements (both neutral and mono-element cations and anions), intends to fill the gap in theoretical and quantum chemistry associated with these substances, which undoubtedly deserve the close attention of various researchers. The author of this preface (and concurrently, the Guest Editor of the given Special Issue (*see photo presented below*)) hopes that the creation of this Special Issue will find wide support from its readers and will contribute to the emergence of new ideas and materials, and valuable comments and suggestions, in this scientific direction.

